# Psychoanalysis in times of plurality of sciences illustrated by the LAC depression study and the MODE study

**DOI:** 10.3389/fpsyg.2026.1778857

**Published:** 2026-03-26

**Authors:** Marianne Leuzinger-Bohleber

**Affiliations:** Poliklinik für Psychosomatische Medizin und Psychotehrapie, Johannes Gutenberg University Mainz, Mainz, Germany

**Keywords:** clinical psychoanalytic research, cognitive behavioral, comparative outcome study, extra-clinical research, LAC study, MODE study, psychoanalytic long-term psychotherapy

## Abstract

From the outset, psychoanalysis has understood itself as a science of the unconscious and has developed specific methods for exploring unconscious fantasies and conflicts. However, even for psychoanalysis, the days of a unified science have passed: contemporary psychoanalysis can be characterized by a pluralism of research methods, theories, and treatment concepts. Concrete examples from the research practice in two large, multicenter therapy outcome studies, the LAC Depression Study and the ongoing MODE study, illustrate the wealth of different research approaches: clinical-psychoanalytic, conceptual, empirical, and interdisciplinary research methods. A graphic is used to show how these different research tools can be fruitfully combined. As will be discussed, contemporary psychoanalysis has developed a stable scientific identity in the midst of contradictions and challenges. Due to this, it no longer has to fear, as Freud did, that it would be “swallowed up by medicine.” Rather, psychoanalytic researchers can feel enriched and stimulated by interdisciplinary exchange with medicine, psychiatry, neuroscience, and many other scientific disciplines. Instead of retreating defensively into an ivory tower, scientific dialogues can be carried out with curiosity and an expectation that they not only open doors to global, intergenerational networks but also to innovative developments within psychoanalysis itself.

## 1 Introduction

Contemporary psychoanalysis still has to fight against the prejudice that it refuses to subject the outcomes of its treatments to empirical evaluations and thus resembles a religion rather than a science. As Whitebook (2017) meticulously discusses in his latest biography of Freud, this is a fundamental misunderstanding. Already Freud himself understood and defined psychoanalysis as a science, albeit one with research topic that is difficult to investigate empirically, namely unconscious fantasies and psychological conflicts. Nevertheless, from the very beginning, some psychoanalysts have attempted to examine the outcomes of psychoanalysis from the point of view of external, empirical observations (see, among others, Wallerstein, 2001; Leuzinger-Bohleber et al., 2020a,b). At the same time, psychoanalysis has always seen itself not only as a treatment method closely linked to the tradition of the Enlightenment, but also as a critical cultural theory, with close ties to philosophy, art, and literature. In the context of research, psychoanalysis often finds itself as trapped between medico-psychotherapeutic and cultural theories, nomothetic and hermeneutic sciences, or between natural science and the humanities.

Personally, I experienced these tensions in a particularly dramatic way during my time as director of the Sigmund Freud Institute (SFI) from 2001 to 2016. As described in detail in another paper, the SFI is inextricably linked to German history and the Holocaust (Leuzinger-Bohleber and Plänkers, 2019). In the late 1950s the directors of the Institute for Social Research in Frankfurt, Theodor W. Adorno and Max Horkheimer, convinced the Hessian Prime Minister, August Zinn, to establish a SFI as a gesture of political reparation of the scathed “Jewish science: psychoanalysis.” The fact that psychoanalysis was near-obliterated by the National Socialists was highlighted in a series of lectures on the occasion of Freud’s birthday, 1956, by renowned emigrated psychoanalysts such as Erik Erikson, René Spitz, Franz Alexander, Michael Balint, and Hans Zulliger. Zinn and other politicians listened to these lectures, which were organized by Alexander Mitscherlich and the Institute for Social Research in Frankfurt.

When Rolf Haubl and I took over the leadership of the SFI in 2002, the *zeitgeist* and the position of psychoanalysis had changed significantly. This change led to a—to us totally unexpected—crisis in 2003: the institute was to be closed. Fortunately, thanks to the enormous efforts of our team and many supporters from national and international psychoanalysis, the closing of the institute was prevented. Today, the SFI is once again firmly anchored in the institutional scientific and psychoanalytic landscape. However, the existential crisis in 2003 forced us to radically rethink psychoanalytic research and to expanding our work to comprise large empirical quantitative studies. Research conducted by scientists from the Freud Institute, was now evaluated with the same criteria as those applied to other scientific institutions, in spite of the weighty cultural tradition of our renowned institute.

It was clear to us, however, that the SFI, at that time the only state-supported research institute in Germany, had to participate in the empirical evaluation of the outcome of psychoanalysis—this was a topical health-policy question at the time. A large comparison study on the outcomes of long-term psychoanalytic and behavioral therapy for chronically depressed patients (the LAC study) was one consequence of this political reality. However, as I shall enlarge on in this paper, it was also important for us—within the randomized prospective therapy-comparison study we envisioned—to creatively address the aforementioned epistemological tension: to satisfy the methodological demands of evidence-based medicine without disregarding the philosophical ideals that the Freud Institute was intended to honor. Our broad ambition to follow seemingly contrary scientific ideals and, as it were, serve different masters, led to intense debates within our own psychoanalytically oriented group as well as in external, interdisciplinary dialogues. Below, I shall summarize some of the insights gained in this endeavor and illustrate them with some of our concrete experiences.

In what follows, I shall, however, first address some general aspects of (a) clinical psychoanalytic research and (b) extra-clinical research. In so doing, I will present the comparative outcome study of cognitive behavioral and psychoanalytic long-term psychotherapy, the LAC study, and the still ongoing Multi-Level Outcome Study of Psychoanalyses of Chronically Depressed Patients with Early Trauma or MODE study.

I begin with some remarks on the history of psychoanalysis within the world of sciences (Section 2). This will be related to some general developments in science at large (Section 3). My aim is to illustrate that the societal changes during the period at hand have strongly influenced the self-understanding of psychoanalysis as a science. It has led to the development of a plurality of different research strategies within our field (Section 4).

## 2 Psychoanalysis in the world of sciences: an eventful history

What did Freud mean when he defined psychoanalysis as a specific “*science of the unconscious*”? As is well known, Freud was interested in philosophy and the humanities before turning to the natural sciences. He worked with medical-neurological research in the laboratory at Ernst Brücke’s Physiological Institute, where he became acquainted with a strictly positivist understanding of science. This form of science attracted him to the end of his life. However, as we know, Freud also turned away from the neurology of his time, as he recognized the limitations of this discipline for investigating unconscious mental processes. With the *Interpretation of Dreams*, the birth document of psychoanalysis, he defined his new science as *pure* psychology—while at the same time continuing to see himself as a scientifically prudent physician. According to Whitebook (2010, 2017), the ideal of an exact, empirical examination of his hypotheses and theories protected Freud from his tendency toward wild speculation. Whitebook characterized Freud as a philosophical physician whose firmly grounded scientific attitude enabled him to establish a new *science of the unconscious*.

In retrospect, this self-understanding is a key to the success story of psychoanalysis, as it guaranteed the independent development of psychoanalysis as a scientific discipline (see, e.g., Makari, 2008). As is well known, in 1909, Freud was still considering whether it would be beneficial for the future of his young discipline to join August Forel’s medical organization “Medical Psychology and Psychotherapy” or even the Orden for Ethics and Culture (a kind of religious organization). Fortunately, however, he decided to found his own psychoanalytic organization, the International Psychoanalytical Association (IPA), on New Year’s Eve 1910 (see, e.g., Falzeder, 2010). Establishing this organization ensured the institutional- and methodological-independence of psychoanalysis as a scientific discipline, independent of academia, to which Freud adhered thereafter. He emphasized, for example, that it was not at all desirable for psychoanalysis “to be swallowed up by medicine”, but that “as ‘depth psychology’, the study of the psychic unconscious [could] become indispensable to all the sciences that deal with the history of the development of human culture and its great institutions such as art, religion and social order […]” (Freud, 1926, p. 283). In this context, his extensive case histories, among other writings, are telling. Freud himself noted that they should be read more like literature than the results of technical medical research.

In the 100 years of its history, the specificity of psychoanalytic science has become increasingly distinct. Psychoanalysis has developed a differentiated, independent research method for the investigation of its specific object of research, unconscious fantasies and conflicts. Accordingly, like other disciplines, psychoanalysis now has quality- and truth criteria that can be presented transparently in scientific dialogues with the outside world.

## 3 Transformations in the world of sciences

Over the last 300 years, Western societies have devoted a large part of their resources to acquiring, expanding and testing knowledge. In the last century, the industrial sector has transformed itself into an advanced knowledge society. If psychoanalysis wants to remain in the world of science, it must take note of these historical and sociological transformations within science and society and understand the impact of these developments on psychoanalytic research realities.

A first component of the transformations in the sciences concerns *differentiation*. As Hermann von Helmholtz noted 100 years ago, each individual researcher is increasingly forced to devote himself to ever *narrower* questions using ever *more specific* methods. Today’s scientists are mostly highly specialized experts with very limited knowledge of adjacent fields; the days of the universal genius are gone (von Helmholtz, 1896, cited by Weingart et al., 2002, p. 703). When contemporary scientists investigate complex problems, they are dependent on international, intergenerational and interdisciplinary networking.

Linked to this process of differentiation, the criteria of science and scientific truths have become more specific and nuanced in both the natural sciences and the humanities. The idea of a unified *Science*, based on the experimental design of classical physics (and the double-blind experiment that emerged from it), proved to be a myth; we live in a time of the *plurality of sciences* (see, e.g., Hampe, 2003; Guggenheim et al., 2016; Leuzinger-Bohleber, 2021).

A second feature of the contemporary transformations concerns the *relationship between science and society*: today’s scientific disciplines—and thus also psychoanalysis—are in constant, accelerated, global competition at various levels. For example, the practical relevance of research findings is constantly evaluated by sponsors and political interest groups that, moreover, have an impact on the funding of research projects. In this sense, science is increasingly losing its self-control. *Science is being politicized—politics is being* scientized.

Due to these developments, the communication of methods and results of scientific studies is absolutely crucial, as is the ability to summarize them in narratives that are understandable to lay people. Albert Einstein was a great storyteller; without this ability, it is doubtful whether the theory of relativity would have had such a wide impact. The same applies to Sigmund Freud and to today’s talented and well-known scientific and psychoanalytical personalities—just think of the Nobel Prize winner Eric Kandel, the neurologist and psychiatrist Vittorio Gallese, the founder of neuropsychoanalysis, Mark Solms, or the probably best-known promoter of mentalization theory, Peter Fonagy, to mention just a few. They are all brilliant storytellers.

A *third characteristic* of the contemporary transformations of science is related to the following: because politics and society expect recommendations for solving social problems more and more quickly, there is less and less time for basic science, from which—after intensive research—reliable and applicable knowledge to be derived. This trend leads to a paradoxical situation: on the one hand, fewer and fewer “normal citizens” and politicians have the confidence to make their own judgment on complex issues without first consulting scientific experts; on the other hand, it has now become common knowledge that even scientific experts often do not present “objective” truths, but that so-called scientific knowledge must always be viewed critically. This, in its turn, involves new risks, as the financial and climate crises as well as the COVID-19 pandemic have revealed. During the pandemic, different leading virologists contradicted each other, especially during its first months, on issues of relevance to individuals and society. For example, the Robert Koch Institute in Germany announced at the end of January 2020 that the risk posed by the “Chinese virus” was considered to be low for the German population (Schnibben and Schraven, 2020). Such experiences with scientific experts are a new source of uncertainty that generates fear. Which scientist is most likely to be trusted depends largely on their *credibility as conveyed by the media*, which are therefore an increasingly critical arbiter of different “truths” in politics and the public sphere.

*A fourth factor in the contemporary development of social and psychological science is therefore the role of the media*. Scientific knowledge is generally only taken note of if it finds its way into the media—and simplified and dramatized accordingly.

“It is a paradox—the more independent science and the media are from each other, the closer their link is. And the more the media gain in importance, the more the sciences lose their monopoly in the assessment of scientific findings. The abstract criterion of truth is no longer sufficient in public debates because the media add the criterion of public acceptance. However, this does not mean that scientific verifications are being replaced, but they are being supplemented by other measures. […] However, this loss of distance [between science and the media, M.L.-B.] will not mean that truths will no longer be communicated. Truth and trust remain both constitutive and rare values in communication, and the more society depends on reliable knowledge, the more it needs it. *The most important characteristic of today’s societies is the competition for trust.* When trust has been earned, it is priceless and science should be very careful not to lose it. Therefore, efforts to produce trust and credibility are becoming ever greater.” (Weingart et al., 2002, p. 706, my translation).

The transformations in the world of sciences outlined above are still underestimated in the psychoanalytical community, even though the influence of the media, especially that of social media has intensified. To what I have outlined so far, one may add the influence from the growing application of Artificial Intelligence. In recent years, and especially since Donald Trump’s second term as President of the United States, the latter two factors in particular have become apparent in a way that was previously unimaginable. Populists and autocratic leaders such as Donald Trump, but also Vladimir Putin and Chinese dictator Xi Jinping, are waging an open battle against scientific institutions, especially if they publish research findings that contradict their political propaganda disseminated in the media. To name just a few of the most obvious current examples: In Russia, this is true for the entire portrayal of the war of aggression against Ukraine; in China the outbreak of the COVID epidemic and in the US numerous issues, such as migration policy and the impact of taxes on the global economy. Donald Trump is waging a power struggle against independent scientific institutions such as Harvard University that was previously unimaginable. In addition, he and the end time fascists who support him (Klein and Taylor, 2025) are undermining trust in science, facts, data, and reality. Hampe (in press) sees this as a sign that the Age of Enlightenment may be coming to an end.

## 4 Some influence of societal changes on psychoanalytic research

What influence have the outlined changes had on psychoanalysis in particular? It seems to me that psychoanalysis, as a science based on the intimacy of the psychoanalytic situation, keenly feels the outlined paradoxes and dilemmas of these transformations. Further, as a science of the unconscious, it appears to be particularly dependent on the trust of the scientific community, the public, politicians and funders, but also on the trust of potential patients, training candidates and health insurance companies. In the 100 years of its history, psychoanalysis has experienced and in part been challenged in many different historical periods and social contexts. Bohleber (2010) has shown this in relation to German psychoanalysis. The dominant zeitgeist has also had an impact on psychoanalysts themselves, including on their understanding of research, its questions, designs and goals. Here are just two examples:

Freud’s lifelong hope was that the time would come when the insights of psychoanalysis (gained through purely psychological/observational methods) could also be objectively tested (by “hard” scientific methods). Today, this seems to be becoming a reality, e.g., through the dialogue with the modern neurosciences.^1^ Forty years ago, however, Habermas (1968), one of the most influential German philosophers, described the Freudian longing for a natural science foundation of psychoanalysis as a *scientistic self-misunderstanding*. He characterized psychoanalysis as pursuing an *emancipatory interest in knowledge*, in contrast to behavioral therapy, which was committed to a *technical interest in knowledge*. This distinction was widely echoed by an entire generation and gave (along with other factors of course) psychoanalysis a heyday that it has never experienced before or since. *Psychoanalysis as a critical-hermeneutic method* of elucidating individual and social contradictions and unconscious sources of psychic and psychosomatic suffering, was met with an exclusive social acceptance that bordered on idealization. Despite the seemingly endless attacks and controversies, psychoanalysis as a treatment method and critical cultural theory did not really have to fear for its existence during this historical period.

This social and philosophical acceptance also shaped the scientific understanding of psychoanalysis. In the 1970s and 1980s, alongside genuine clinical-psychoanalytical research—research carried out within the psychoanalytic situation itself (see Section 5)—hermeneutically oriented approaches and related socio-psychological and cultural-critical analyses flourished, as did interdisciplinary exchanges with philosophy, sociology, humanities and education, as well as studies on film and art. Empirical and particularly quantitative research or dialogues with the natural sciences were, particularly in Germany and France, judged to be naïve and inappropriate for psychoanalysis, even harmful. This had far-reaching consequences even in the US, as Hustveth (2010) laconically states in her bestseller *The Trembling Woman*:

“Although American psychiatry used to be heavily influenced by psychoanalysis, the two disciplines have grown further and further apart, especially since the 1970s. Many psychiatrists know little or nothing about psychoanalysis, which has become increasingly marginalized in our culture. A large number of American psychiatrists prefer to leave the talking to social workers and stick to prescribing. Drug treatment prevails. Yet, many psychoanalysts are still practicing around the world, and it is a discipline that has fascinated me since I first read Freud at sixteen […].” (p. 26)

As Kuhn (1997) puts it in his analyses of the history of science, different paradigms always exist side by side. However, one of them usually dominates—the one that best suits the current zeitgeist. It seems to me that the understanding of psychoanalysis as a critical hermeneutics is still strongly represented in French psychoanalysis and to some extent in Latin America, while we in Anglo-Saxon and German-speaking psychoanalysis often has to fight against over-adaptation to an empirical-quantitative research paradigm (see, e.g., Fonagy et al., 2015; Leuzinger-Bohleber et al., 2020a,b; Leuzinger-Bohleber, 2021). In the last-mentioned countries, the zeitgeist has changed dramatically: In our times of “evidence-based medicine,” the impression can sometimes arise that only one form of research exists for psychoanalysis, as for other medical science, namely empirical-quantitative psychotherapy research, molded in the paradigm of the classical natural sciences. This is—on closer inspection—a strange return of a long outdated and problematical idea of a “unified science” (Hampe, 2003; Guggenheim et al., 2016), an unconscious simplification of the complexities of research in the knowledge society, an outlook that entails, in my opinion, dangers for psychoanalysis.

In connection with the above-mentioned developments of recent years and the increasing hostility toward science in autocratic societies, it is quite possible that new solidarities among scientists all over the world will emerge. Perhaps the great danger posed by fake news and demagogic possibilities in social media and on the internet will lead to a new appreciation of all forms of science. In contrast to fake news and the manipulation of “truths” for political gain, a basic scientific attitude, in the sense of a commitment to verifiable facts and data, is likely to unite representatives of a wide range of scientific paradigms. This also applies to psychoanalysis, where, in my perception, there are signs of increasing acceptance of the *plurality of different research methods in modern psychoanalysis*.^2^

## 5 Psychoanalytic research today

My aim in the following is to briefly discuss the challenging epistemological and methodological problems we have in these times of scientific and methodological pluralism (for more details, see Leuzinger-Bohleber, 2010, 2015a,b; Leuzinger-Bohleber et al., 2020a).^3^ For example, in the large comparative therapy outcome study on chronic depression, the LAC study, as well as in the ongoing MODE study we applied a broad spectrum of current psychoanalytic research strategies. We thus tried to meet the demands of evidence-base medicine without renouncing the independence of psychoanalysis as a scientific discipline for studying unconscious mental processes.

In order to briefly discuss this complex challenge, I would like to refer to a diagram of clinical and extra-clinical psychoanalytic research which I had developed in different earlier papers. The LAC and MODE studies serve as illustrations of my considerations.

### 5.1 Two outcome studies on psychoanalyses

The LAC study: *outcomes of cognitive-behavioral (CBT) and psychoanalytic (PAT) long-term treatments for chronically depressed patients* is the first controlled psychotherapy study to compare the outcomes of psychoanalytic and cognitive-behavioral *long-term* psychotherapy in chronically depressed patients with both randomized and preferred assignment. In this study 554 chronically depressed patients were recruited in Frankfurt am Main, Berlin, Hamburg, and Mainz. Strict inclusion criteria and practical circumstances reduced the sample to 252. Once theses participants had started therapy, the dropout rate was low, regardless of the therapeutic group: after 3 years, 165 patients, i.e. around 65%, were still in the study.

Both psychotherapy methods proved successful in achieving a significant reduction in symptoms in these patients, many of whom had been ill for a long time. The patients’ self-assessments showed major and stable changes. After just 1 year, the initial average BDI^4^ score fell by 12.1 points from 32.1, and after 3 years by as much as 17.2 points. The effect sizes^5^ were very high: *d* = 1.17 after 1 year and *d* = 1.83 after 3 years. The complete remission rate^6^ was 34% already after 1 year and rose to 45% after 3 years. Similar results were found in the assessments by independent raters who were blinded to the treatment methods: QIDS-C^7^ scores decreased from 14.1 to 7.1 in the first year and further to 7.0 3 years after the start of treatment. The effect sizes this measure, too, were high, rising from *d* = 1.56 after 1 year to *d* = 2.08 3 years after the start of treatment. Contrary to our hypotheses concerning possible differences between both treatment groups, however, we found no statistically significant difference between PAT and CBT or between randomized and preferred treatment. Furthermore, CBT was not more effective in reducing the depressive symptoms after 1 year. PAT also led to a statistically significant reduction in symptoms after 1 year. However, contrary to our hypothesis, PAT was not superior to CBT in respect to symptom reduction 3 years after the start of treatment, but equally effective. The remission rates achieved are better than in other studies: 39% of patients showed full remission after just 1 year and 61% after 3 years after the start of treatment. Chronically depressed patients therefore benefit from long-term psychotherapy (Leuzinger-Bohleber et al., 2019a). In a second publication we examined so-called structural changes, which are considered central to sustainable psychological changes in psychoanalysis and long-term psychoanalytic treatment. After 3 years, statistically significantly more patients in PAT (60%) met the criteria for a *structural change* on the Heidelberg Restructuring Scale (Rudolf et al., 2000) compared to CBT (36%). In addition, after 3 years, there was a stronger association between *structural changes* and *reduction in depressive symptoms* in PAT than in CBT (Leuzinger-Bohleber et al., 2019b). Furthermore, in an additional evaluation of the data, we found that PAT is superior to CBT in symptom reduction in traumatized, chronically depressed patients (Krakau et al., 2024).

The *Multi-Level Outcome Study of Psychoanalyses of Chronically Depressed Patients with Early Trauma (MODE)* is a sequel to the LAC depression study, adding brain-imaging methods to the methods applied in LAC. MODE compares the outcomes of low frequency psychoanalysis (one session/week) with high frequent psychoanalysis (3–5 sessions/week) for the treatment of chronically depressed patients who have experienced childhood trauma. It also includes an untreated healthy control group. The major hypothesis of this randomized controlled study is that both manualized interventions will lead to a significant improvement, though the high frequency treatment will produce demonstrably better outcomes in comparison to less frequent psychoanalytic treatment. Treatment progress is assessed in terms of symptom reduction (using standard psychological measures), structural change (defined by psychoanalytic methods), and neurobiological change (assessed using MRI). Centers for the study are in Germany (Frankfurt, Cologne, Leipzig, Gießen, Mainz), the US (Los Angeles/San Francisco) and Switzerland (Lausanne) (see Ambresin et al., 2023).

Recruitment was completed in all centers in May 2024. A total of 80 patients for the MRI examination and 45 control subjects were included. The rest of the 112 psychoanalysis patients could not be assessed with MRI for various reasons (tattoos, retainers, etc.), but remain in the psychological arm of the study.

Conceptual considerations as well as clinical observations of the ongoing MODE study are discussed below.

### 5.2 Overview of the plurality in psychoanalytic research today

#### 5.2.1 Clinical research

Clinical research takes place in the intimacy of the psychoanalytic situation. Most of psychoanalysts worldwide still identify with this form of research, which Freud (1926), as is well known, characterized as the inseparable bond between cure and research (in German: “Junktim zwischen Heilen und Forschen”):

“In psycho-analysis there has existed from the very first an inseparable bond between cure and research. Knowledge brought therapeutic success. It was impossible to treat a patient without learning something new; it was impossible to gain fresh insight without perceiving its beneficent results. Our analytic procedure is the only one in which this precious conjunction is assured.” (p. 255).

From today’s epistemological perspective this form of clinical-field research can be described as a circular process of discovery, of gaining knowledge in which observations of unconscious fantasies and conflicts are successively visualized, symbolized and finally put into words by the analyst in collaboration with the analysand. Every gained insight into the unconscious fantasies and conflicts of the analysand in a given analytic session contributes to shaping our evolving theories, which we—implicitly, at first in the back of our minds—take with us into the next psychoanalytic session. At the same time, the psychoanalytic attitude that we nevertheless try to adopt includes an openness to not knowing, of neutrality, and openness to new perceptions, observations, and ideas—in the sense of evenly floating attention (Freud). This professional attitude is a prerequisite for entering into the circular process of joint exploration and understanding of unconscious processes with the analysand, as outlined on the left side of the graph. The circular processes of discovery initially take place mainly unconsciously and are influenced by implicit, private theories. Only a small part of these processes are accessible to the analyst’s conscious reflection. In a second step, the insights gained in this form of clinical research may be critically discussed within and outside the psychoanalytic community: they become part of the so called “official theories” (see, e.g., Bohleber et al., 2013).

Often, first insights or hypotheses are discussed with colleagues in supervisions or compared with clinical vignettes or specimen cases and hypotheses or theories published in the psychoanalytic literature. This kind of clinical psychoanalytical research and case presentations remains the most common means of communication among psychoanalysts at scientific and clinical conferences as well as in psychoanalytic training. In most psychoanalytic societies training is still concluded with the *presentation of a case presentation*. There are reasons why Guggenheim et al. (2016) characterized psychoanalysis as a *“case science.”*

From an epistemological perspective it is important to note that clinical-psychoanalytic research aims at decrypting unconscious meanings of fantasies and conflicts, personal and biographical uniqueness, and various unconscious facets of the specific micro-worlds of the analysands (Moser, 2009). Therefore, clinical research may be characterized as *critical hermeneutics*. It has great similarities with narratives in literature, film and other art forms that also communicate knowledge about complex unconscious processes of the human psyche.

The professionalism of the analyst, the ability to make use of free floating attention as well as psychoanalytic theory, enables him to use his own countertransference reactions, scenic observations of “embodied enactments” (Argelander, 1970; Bohleber and Leuzinger-Bohleber, 2016), “Freudian slips” and not the least dreams as source material for understanding the unconscious psychodynamics of the analysand.

It is important to underscore that the psychoanalytic research process of decoding “unconscious truths” can only be carried out together with the analysand. This is another central, characteristic feature of psychoanalysis—in contrast to the top-down approach of behavioral therapy, for example. Linked to this is the characteristic “truth criterion” of psychoanalytic interpretations: Whether a particular interpretation of unconscious fantasies or conflicts is “true” can only be judged *together* with the analysand as in the joint observation of his (unconscious and conscious) reactions to an interpretation.

The majority of all the insights gained in psychoanalysis in the last 100 years are indeed based on the described intensive and meticulous “field observations” with individual patients in the analytic situation (see also Kleinian tradition of psychoanalysis, e.g., Britton, 2009; Anderson, 2014).^8^ Furthermore, von Braun and Held (2025) see clinical research in psychoanalysis as a unique opportunity to study profound cultural transformations and their influences on the unconscious of individuals. The results of clinical research in psychoanalysis may thus contribute to a critical analysis of culture and society.

Nevertheless, to avoid any misunderstandings: Peter Fonagy is probably right when he points out that not every clinician is automatically a researcher. Only a methodologically systematic approach makes clinical observations accessible to the understanding and criticism of a third party, and thus makes them open to criticism from outside, which for many authors is a fundamental criterion of science (“Wissenschaft”) as opposed to art.

It is well known that we in psychoanalysis, more than almost any other clinical discipline, have a differentiated culture of clinical conferences and supervision groups. These groups offer the possibility to critically reflect, together with others, on clinical observations. This specific tradition can be used for psychoanalytic research, e.g., by applying the so called Three-Level of Clinical Observations method, developed by a subcommittee of the Research Committee of the IPA (see, e.g., Hanly et al., 2021). This method was an attempt to cope with the problems of subjectively selected clinical vignettes, chosen to illustrate theoretical concepts, instead of verifying and critically developing these concepts further. Too few psychoanalytic concepts are critically linked to—confirmed or refuted by—outcomes of extra-clinical research. Also, relatively few efforts have been made so far to counter the well-known lack of detail in case presentations with self-critical alternative interpretations (with questions about selection and condensation of the clinical observations, or the issue of the writer’s blind spots). In addition to the increasingly difficult problem of data protection, this is probably one of the main reasons why extensive case presentations are increasingly rare in international psychoanalytic journals, even though, as explained above, they are a central component of clinical psychoanalytic research.

*Since we are convinced that we urgently need good clinical research, not only to survive in the world of psychotherapies, but also to constantly develop our professional art of treatment (see, e.g., Kernberg,* *2006**; Colombo and Michels,* *2007**; Leuzinger-Bohleber,* *2017**), we have, in the context of the LAC depression study, taken up the tradition of detailed case reports. We used our own form of clinical research by discussing the treatments (some of which were tape recorded), in weekly “clinical conferences” (which also were systematically documented). In line with the above mentioned Three-Level-of Clinical Observation approach we discussed single cases systematically and applied the method of so called expert validation (see Leuzinger-Bohleber et al.,* *2003**). In our view, this was merely a first step of a systematic investigation. The results are documented in narrative case reports, which are published (Leuzinger-Bohleber et al.,* *2020b**; Leuzinger-Bohleber,* *2015a**,**b**). In our own assessment these extended case reports may are an important outcome of the LAC study. We hope they are l helpful in the dissemination of psychoanalytic insights into the specific psychodynamics of chronic depression and that they will shed light on its complex individual and cultural determinants as well as on adequate treatment technique.*^9^

#### 5.2.2 Conceptual research

All forms of clinical research may become part of *psychoanalytic conceptual research*, which is as old as psychoanalysis itself. The continuous development and advancement of concepts have always been intrinsic in psychoanalysis and contributed to its appeal to intellectuals, writers, artists and researchers from other disciplines.

In the 1990s, Joseph Sandler and Anna Ursula Dreher nonetheless presented a new definition of psychoanalytic conceptual research and differentiated it from other forms of research. And so, in the Research Subcommittee for Conceptual Research we tried to further specify and differentiate conceptual research, but also to clarify quality criteria for this specific field (for details see Leuzinger-Bohleber and Fischmann, 2006). Another approach to conceptual research, published by the IPA Committee for Conceptual Integration chaired by W. Bohleber—with contributions from P. Fonagy, D. Scarfone, J.P. Jimenez, P. Denis, D. Zysman and others—discussed developments of central psychoanalytic concepts like unconscious phantasy or enactment (Bohleber et al., 2013).

*The aforementioned form of clinical psychoanalytic research in the LAC study was closely linked to conceptual research. Individual papers, e.g., on the unconscious determinants of chronic depression and the significance of trauma, were published, e.g., discussing specific treatment questions based on clinical as well as conceptual research on trauma and depression (e.g., Bohleber and Leuzinger-Bohleber,* *2016**; Leuzinger-Bohleber,* *2015a**,**b**,* *2025**). All conceptual papers have been collected in a kind of textbook on psychoanalytic treatment of chronically depressed patients (Leuzinger-Bohleber et al.,* *2022**). This “manual” was used to train the therapists in the MODE study (see Ambresin et al.,* *2023**).*

#### 5.2.3 Extraclinical research

**Figure 1:**
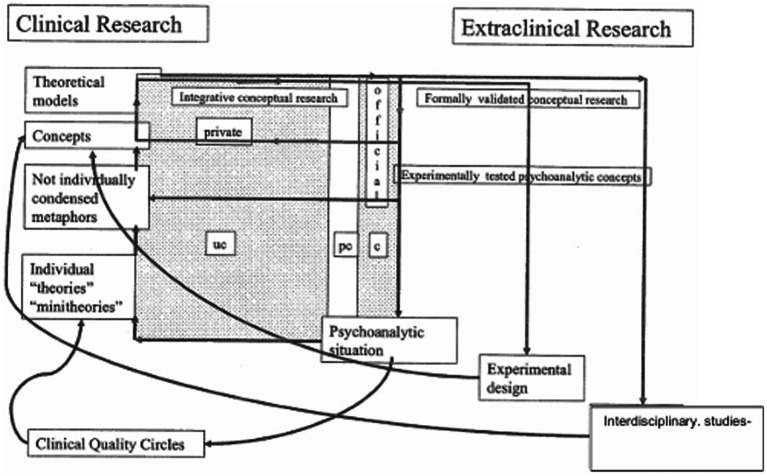
Explanation of the research pathways in the graph.

The results of both clinical-psychoanalytic and conceptual research can become the object of further *extraclinical research* (see Figure 1). We distinguish between *empirical*, *experimental* and *interdisciplinary* studies.

As mentioned above, psychoanalytic psychotherapy research—one of the *extraclinical empirical research fields (B 2.1)*—has proven to be indispensable in today’s knowledge society. In the current utilitarian zeitgeist it is essential to prove the effectiveness of psychoanalytic treatments. It is, further, important to do so in accordance with the criteria of “evidence-based medicine.”

*In the LAC study, the outcomes (achieved with methods that meet these criteria) were therefore published in renowned international journals (see summary, and, e.g., Leuzinger-Bohleber et al.,* *2019a**,**b**; Krakau et al.,* *2024**).*

Reviewing the history of psychoanalytic empirical psychotherapy research Wallerstein (2001) traces its beginnings to 1917. A large number of outcome studies are now available. Thanks to new techniques (video/audio recordings and digitalization), many of today’s studies combine microanalyses of therapeutic processes with outcome research.^10^ Thorough extraclinical research always involves a great deal of effort, which can only be accomplished in an appropriately equipped network within research institutions. They are always connected with challenging dependencies between the generations of researchers involved, as we discussed in connection with the LAC study (Leuzinger-Bohleber and Plänkers, 2019).

Within *experimental psychoanalytic studies (B 2.2)* one has to face that one cannot, in an experimental design, directly test psychoanalytic processes. Unconscious mental processes are rarely possible to operationalize so precisely that they can be tested in a research laboratory.

*However, some research groups, such the group conducting the MODE study, are attempting to study patients in a randomized, controlled design, using, among other things, neuroscientific methods (the MRI scanner). Such studies of quantitatively measurable outcomes of psychoanalyses can certainly be considered experimental. Other research groups have experimentally investigated psychoanalytic concepts directly, for example, preconscious and unconscious information processing in memory and dreams (e.g., Ellman and Antrobus,* *1991**; Hau,* *2008**; Leuzinger-Bohleber et al.,* *2008**).*

Finally, we would like to mention the broad field of *interdisciplinary studies* (B.2.2.3). In recent years, the dialogue with the neurosciences has opened new doors for psychoanalysis and its research. To mention just one example, international research teams of the network of the Society for Neuro-psychoanalysis, recently founded by Mark Solms, are conducting a wealth of experimental FmRI and EEG studies on psychoanalytical issues (e.g., at the University hospital in Zurich, at the Centre Hopitalier Universitaire Vaudois (CHUV) in Lausanne or at the University College London.

*As mentioned above: In the MODE study, we have an intensive dialogue between psychoanalysts and neuroscientists. Changes measured by brain imaging methods were chosen as the primary outcome criterion for the study. Interdisciplinary dialogues are also prominent in many sub-studies of MODE. To mention just one example: one psychoanalytically form of data collection is the procuring dream diaries from participating patients. These diaries are evaluated with various methods. Dream diaries are, for example, compared with dream narratives that were told in psychoanalytic sessions. We have presented some initial papers on this topic at various psychoanalytic conferences, including the ApsaA conference in San Francisco in February 2025 and the IPA Congress in Lisbon in July 2025. Furthermore, the dream diaries are investigated with the help of a range of extraclinical (psychological) instruments such as the Linguistic Inquiry and Word Count (LIWC) and DREAMS-C, a scale developed by our research group in Frankfurt (led by Tamara Fischmann) to study transformations of manifest dreams during psychoanalyses (cf. Leuzinger-Bohleber et al.,* *2023**; Ambresin and Leuzinger-Bohleber,* *2024**). In addition, the working group is developing the Zurich Dream Process Coding System (ZDPCS), which instrument takes into account findings from contemporary experimental and neuroscientific research on dreams (see, e.g., Fischmann and Leuzinger-Bohleber,* *2025**).*

However, we would like to mention that not only *interdisciplinary studies* in the field of neuropsychoanalysis are crucial for the acceptance of psychoanalysis in today’s world of science. We also need the creative exchange with, for example, attachment research, empirical developmental research, and embodied cognitive science. Equally important is interdisciplinary research in cooperation with literary and cultural studies (see, e.g., Von Hoff, 2025), social psychology, philosophy, media and communication studies as well as ethnopsychoanalysis.

## 6 Summary and conclusion

In this paper we tried to shed light on some of the distinctive features of psychoanalytic research. First, we provided a brief overview of its scientific history. As is well known, Freud placed psychoanalysis within the natural sciences, but at the same time he emphasized its proximity to philosophy, the humanities, literature, and art. This led to many debates about the specificities of psychoanalysis as a science. Lorenzer’s (1970) formulation of psychoanalysis as a “science between the sciences,” between the natural sciences and the humanities, biology and culture, is borne out by the outlined development.

However, as briefly discussed in Section 4 of this paper, psychoanalysis, like other scientific disciplines, has undergone a continuous development over the last 100 years. The idea of a unified psychological science (based on the model of classical physics) is a thing of the past: we live in an age of pluralism of sciences. Even in physics today, there is a pluralism of different, highly specialized methods that are used to investigate very specific research questions. Against this backdrop, psychoanalysis is no longer a unique, somehow weird case: psychoanalysis, as all other scientific disciplines, has developed specific methods to investigate its specific research object. Its results are evaluated according to specific quality criteria, and they are critically discussed in psychoanalytic and interdisciplinary discourses. To put it somewhat simplistically—Psychoanalysis, as a scientific discipline, has now found a more stable and self-confident self-understanding. Perhaps one could talk about a stable scientific identity in the midst of contradictions and challenges. Due to this, it no longer has to fear, as Freud did, that it would be “swallowed up by medicine” (see Section 2). Rather, in contemporary psychoanalysis, we can feel enriched and stimulated by interdisciplinary exchange with medicine, psychiatry, neuroscience, and many other scientific disciplines. Instead of retreating defensively into an ivory tower, scientific dialogues should, in our view, be carried out with curiosity and an expectation that they not only open doors to global, intergenerational networks—without which, according to the sociologists around Peter Weingart (see Section 3), contemporary science would be inconceivable—but also to innovative developments within psychoanalysis itself.

In the main part of this paper (Section 5), we have tried to illustrate that today’s psychoanalysis has a richness of diverse research methods for investigating the unconscious. We can build bridges between clinical, conceptual, and extra-clinical (i.e., empirical, experimental, and interdisciplinary) research. Two large studies, the LAC Depression Study and the ongoing MODE study, illustrate possibilities for a combination of different psychoanalytical research strategies with which we may investigate complex research questions from different perspectives that may complement, correct and relativize each other.

Today, many populists and autocrats are attacking the recognition of realities and facts. This is a threat to the basis of democracy as well as every form of science. We hope that a new awareness will emerge that all researchers and scientists, including psychoanalysts, are in the same boat.

## Author’s note

This text is based on earlier German publications, including my introduction in the book: *Was nur erzählt und nicht gemessen werden kann* (Leuzinger-Bohleber et al., 2020b) and a paper in the German journal of philosophy (Leuzinger-Bohleber, 2021). Other summaries of the history of psychoanalysis (see introduction of this paper) have been published in Leuzinger-Bohleber et al. (2020a) and Leuzinger-Bohleber (2023).

## Footnotes

1 To mention just one example: in the MODE study (see Section 5) methods like brain imaging, in combination with psychological and psychoanalytical instruments are applied in order to investigate the outcomes of psychoanalyses.

2 I do not deny that there is still considerable resistance to empirical research within psychoanalytic communities. On the other hand, I see it as a new indicator for the acceptance of research that the Board of the International Psychoanalytic Association approached the Research Committee in 2017 with the request to make the politically controversial question of the influence of the reduction of weekly frequency of psychoanalysis on the outcome of the treatment the subject of an empirical study. The large multi-level outcome study of psychoanalysis for chronically depressed patients with early trauma study MODE currently underway has taken up this request and is conducting such a study (see Ambresin et al., 2023). An even more recent example is that the IPA Board approached the Research Committee (RC) 2024 to initiate a research project investigating the question of a meaningful integration of tele-analysis into psychoanalytic training. In cooperation with the RC, an Action Research Project was carried out in the summer of 2025 (see Leuzinger-Bohleber et al., in press).

3 The main aim of this paper is to highlight the diversity and plurality of contemporary psychoanalytic research. Illustrations are drawn primarily from the author’s own research projects, as any attempt to address the many different studies in this field would exceed the limited scope of this article.

4 BDI, Beck depression inventory. This was one of the main instruments used to measure symptom reduction (assessed by the patients themselves).

5 Effect sizes are a measure used to compare the effectiveness of different studies. According to Cohen (1988), an effect size of 0.2 is considered small, 0.5 is medium, and 0.8 is high. The effect sizes in the LAC study are therefore surprisingly high.

6 Complete remission means that patients no longer have higher symptom scores than a healthy control group.

7 QIDS-C: Inventory of depressive symptomatology. This was the second main instrument used to measure symptom reduction (assessed by independent raters).

8 Kris (1951), characterized psychoanalysis as a whole, decades ago, as *action research*, because the so called “Junktim Research” in psychoanalysis (the combination of healing and researching in the psychoanalytic situation) could be characterized as a kind of participatory field research that is typical for the so called action research developed by Kurt Lewin in 1940.

9 The illustrations of the conceptual considerations with the help of comments on the LAC or depression study are highlighted here in smaller font and italics in the text.

10 See, e.g., Emde and Fonagy (1997); Kächele et al. (2006); Leuzinger-Bohleber (2010, 2015a,b), Open Door Review (Leuzinger-Bohleber and Kächele, 2015), including the excellent overview by Fonagy; for more recent studies on long-term treatment, see Leichsenring and Rabung (2008), Leuzinger-Bohleber et al. (2020a).
